# Bone Marrow Aspirate Clot: A Useful Technique in Diagnosis and Follow-Up of Hematological Disorders

**DOI:** 10.1155/2019/7590948

**Published:** 2019-03-10

**Authors:** Lucas Oliveira Cantadori, Rafael Dezen Gaiolla, Ligia Niero-Melo, Cristiano Claudino Oliveira

**Affiliations:** ^1^São Paulo State University (UNESP), Medical School of Botucatu, Department of Internal Medicine, Botucatu, SP, Brazil; ^2^São Paulo State University (UNESP), Medical School of Botucatu, Department of Pathology, Botucatu, SP, Brazil; ^3^São Luiz/D'or Hospital, Department of Pathology, São Paulo, SP, Brazil

## Abstract

Bone marrow biopsy is a diagnostic tool largely used in the evaluation of a broad number of disorders that could affect the hematopoietic system. Differently, bone marrow aspirate clot technique is rarely performed even though it has been described in literature. Here, we highlight the utility of the bone marrow aspirate clot, exemplifying through the discussion of three clinical cases in which this technique was used for diagnosis and follow-up purposes: megaloblastic hemopathy, multiple myeloma, and chronic lymphocytic leukemia. Bone marrow clot analysis increases sensitivity to diagnose hemopathies and offers the possibility of morphological evaluation and anatomopathological study, with the advantage of not needing decalcification processes, hence improving antigenic expression in immunohistochemical and FISH techniques. It is an easy-to-perform technique, offering a quick, reliable, and more comfortable procedure for patients.

## 1. Introduction

Bone marrow (BM) evaluation is essential for the diagnosis of primary hemopathies, neoplastic or not, and other diseases that may occur with hematological repercussion. Moreover, this analysis is mandatory for lymphoma staging and can also provide material for analysis of potential prognostic and predictive factors that could drive treatment in specific diseases. BM evaluation is important, for example, for the setting of cytogenetic and molecular profile in acute leukemia or multiple myeloma and the continuous assessment of these pathologies [1, 2].

The procedure usually results in an aspirate sample and a bone fragment sample (trephine biopsy). A smear (myelogram) is obtained from the aspirate for a qualitative and quantitative cytological assessment of BM cells. This material can also be used for flow cytometry, conventional and molecular cytogenetics, and microbiological exam when infections are suspected. The bone fragment is technically processed for histopathologic and immunophenotypic analysis by immunohistochemistry (IHC) allowing not only the morphological assessment but also an analysis of BM architecture, based mainly on the relationships established between the hematopoietic tissue and bone trabeculae and central sinusoids and the grade of extracellular matrix deposition. [2, 3].

It is important to point out that, in Brazil, the analysis of the myelogram is restricted to hematologists, whereas the analysis of the trephine biopsy (bone fragment) is a pathologist's responsibility.

After performing the myelogram, the resulting clot from this procedure is commonly discarded although it may also be utilized for histopathological evaluation with IHC and molecular research studies, for example, *in situ* hybridization technique [4, 5], which allows, therefore, the histological evaluation to supplement the cytological analysis and broadens exam sensitivity.

BM aspirate clot (BMC) technique is not frequently utilized in medical practice. Even though it has been described in the literature, few studies evaluated its applicability in the diagnosis and follow-up of several hematological diseases.

In this article, the technique to make BMC will be described and its practical applicability will be shown through the description of three clinical cases.

## 2. Materials and Methods

### 2.1. Trephine Biopsy and Bone Marrow Aspirate Technique

Bone marrow biopsy is done in the posterior iliac crest while the patient is in right or left lateral decubitus position. In this procedure, sedation is optional. After asepsis and antisepsis, local anesthesia with xylocaine 1% without a vasoconstrictor is applied. The most used needle is an 11-*gauge* Jamshidi needle which is inserted perpendicularly to the bone surface from the posterior iliac crest and directed to the anterior iliac crest, avoiding the needle penetration in the sacroiliac component. The aim is to collect a bone fragment that is approximately 1.5 cm long, which is firstly stored in a flask with buffer formaldehyde at 10% and then processed with a decalcification period which is a critical step to guarantee materials with conditions for evaluation and utilization of complementary techniques [1].

Bone marrow aspirate for myelogram and aspirate clot is done preferably in the sternum manubrium (in adults) with the patient in dorsal decubitus position. However, caution should be taken in cases of multiple myeloma due to the higher risk of sternum perforation. In these cases, the aspiration is preferably done in the posterior iliac crest, right before the bone marrow biopsy. Sedation is usually not needed. After asepsis and antisepsis, local anesthesia with xylocaine 1% without a vasoconstrictor is applied. There are several needles available in the market, all disposable ones. The currently utilized model in our service is an 18-*gauge* needle by 90 millimeters (18G × 90 mm). The needle is inserted perpendicularly to the medium point of the sternum manubrium, 1 cm above the angle of Louis. After needle fixation, the spindle is removed to couple a 5-ml syringe and aspirate the maximum amount from 1 to 2 ml of bone marrow, avoiding sample hemodilution. Next, the smear is prepared for a total of 6 slides, and the rest of the exceeding material is separated on an extra slide until coagulation. If extra material is needed for other exams (BM karyotype, for instance), a new syringe is coupled to the needle and aspiration is done without new punctures.

### 2.2. Bone Marrow Aspirate Clot Processing Technique

**Figure 1:**
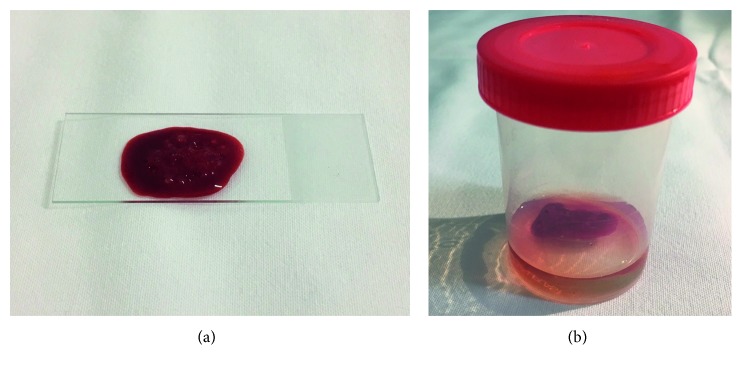
Sample of bone marrow clot after bone marrow aspirate harvest. (a) Material after harvest placed on a slide. (b) After clot formation, the material is fixed in buffer formaldehyde at 10% and processed conventionally with the advantage of dismissing decalcification.

After a bone marrow aspirate, the material left on the extra slide must be placed, after coagulation, in a flask with buffer formaldehyde at 10% or Bouin liquid similarly to the procedures in other biopsy handlings (Figure 1). The technique is quite similar to the cell-block procedure after fine-needle aspiration.

Once the material is fixed, the same procedure for a common biopsy is followed. Briefly, it is measured in macroscopy, placed in a cassette and submitted to five alcohol baths, followed by five xylol baths and, finally, impregnated with paraffin. The whole procedure is done overnight. Afterwards, the sample is removed from the cassette and immersed in liquid paraffin at 60°C, forming a paraffin block. Slides are made from 3- to 4-millimicron cuts of this block.

The cuts are stained according to the standard staining of our service which is hematoxylin and eosin (H&E), Perls (or prussian blue stain), and reticulin. It is noteworthy to point out that reticulin staining, even without the presence of bone support, results in excellent-quality slides for visualization and quantification. Specific immunohistochemical staining is carried out accordingly when indicated.

### 2.3. Clinical Cases

In order to exemplify the clinical applicability of the described technique, samples of bone marrow clot at the time of the diagnosis of three different patients attended in the Hematology Service of the Clinical Hospital of Botucatu Medical School, UNESP, in Brazil, will be presented.

In all three patients, both the diagnosis and follow-up assessment were based only on bone marrow aspirate clot technique.

## 3. Results

### 3.1. Case 1

**Figure 2:**
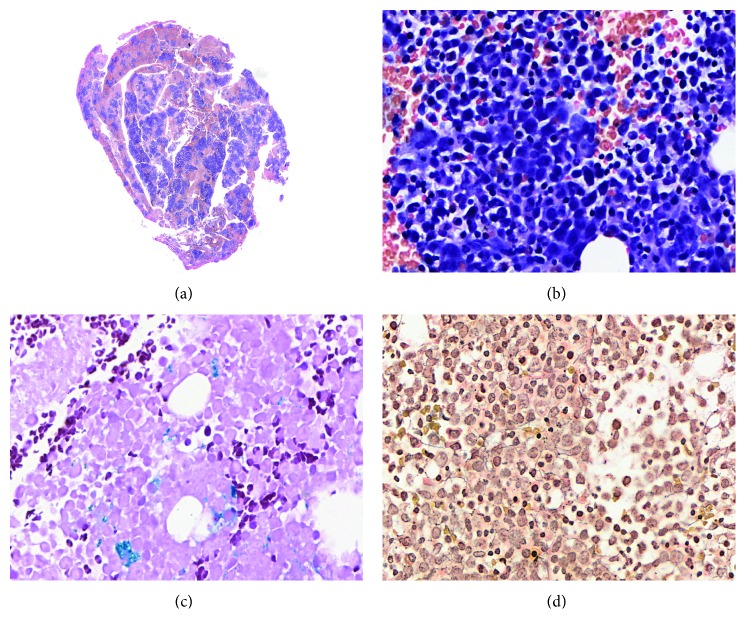
Case 1. The patient with final diagnosis of megaloblastic hemopathy (pernicious anemia). (a) Microscopic panoramic image to show the general aspect of BMC (H&E, 10x). Each grouping of hematopoietic material is called spicule. Five spicules are necessary to consider a material representative for analysis. The utilized criteria to render MC are the same used to interpret BMB. (b) Erythroid hyperplasia with numerous megaloblasts (H&E, 400x). (c) Demonstration of histochemical staining application on MC (Perls, 400x). In the image, hemosiderin deposits are stained blue. (d) Application of reticulin histochemical staining on MC (reticulin, 400x). Reticulin fibers are stained black. It is possible to evaluate this network with this technique similarly to BMB.

A 78-year-old woman was referred from the internal medicine due to symptoms of progressive fatigue, tiredness at small efforts, and intermittent claudication. During physical exam, she presented paleness and atrophic glossitis. Hemogram with pancytopenia and macrocytosis and high lactate dehydrogenase (LDH) (Figure 2).

### 3.2. Case 2

**Figure 3:**
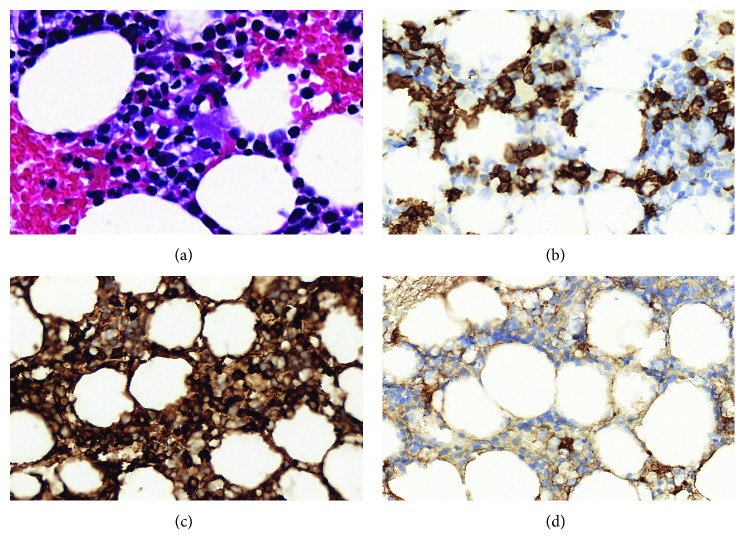
Case 2. The patient with final diagnosis of multiple myeloma. (a) Histological section showing a small grouping of plasmocytes in the middle (H&E, 400x). (b) Utilization of IHC to detect, confirm, and quantify plasmocytes (IHQ, CD138, 400x). Resource applied for diagnosis and posttreatment reevaluations. IHC evaluation confirming that plasmocytes present restriction of immunoglobulin light kappa chain, indicating the diagnosis of multiple myeloma: (c) (kappa, 400x) and (d) (lambda, 400x).

An 87-year-old man was checked in the emergency room presenting symptoms of mental confusion, tiredness at small efforts, and intense lumbar pain. During physical exam, he was clumsy and dehydrated. Lab exams showed he had anemia, hypercalcemia, and renal insufficiency. X-rays showed multiple lytic lesions in the axial skeleton (Figure 3).

### 3.3. Case 3

**Figure 4:**
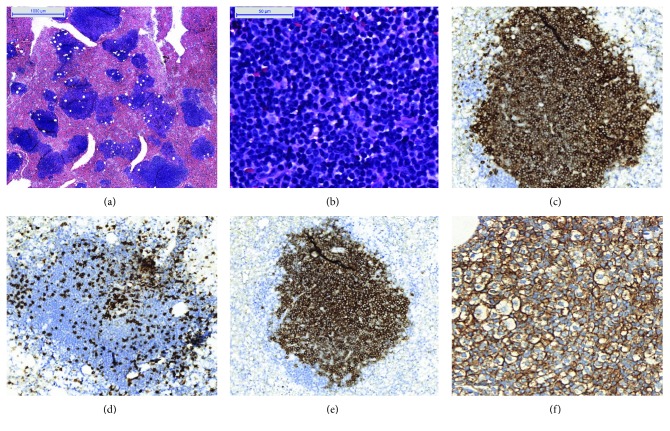
Case 3. The patient with final diagnosis of chronic lymphocytic leukemia (CLL). (a) BMC, in small magnification, showing intense hypercellularity of bone marrow with lymphoid accumulation (H&E, 40x). (b) Cell details, characterizing lymphocytes as small and typical of this neoplasia with hyperchromatic nuclei and regular membrane (H&E, 400x). (c) Immunopositivity for CD20, confirming B-cell origin of the neoplasia (IHC, CD20, 200x). (d) Contrast in lymphoid accumulation, showing a small percentage of T lymphocytes (IHC, CD3, 200x). (e) Immunopositivity overlapping for CD5 in neoplastic B cells (IHC, CD5, 200x). (f) Immunopositivity for CD23 allows diagnosis of CLL (IHQ, CD23, 200x). Note the quality of immunostaining in the images. In BMB samples, due to calcification, color irregularities can compromise analysis and make precise diagnostics sometimes difficult.

This is the case of a 65-year-old man, undergoing clinical follow-up due to lymphocytosis and thrombocytopenia in routine exams, and is asymptomatic (Figure 4).

## 4. Discussion

Conventional trephine bone marrow biopsies have bone trabeculae, requiring material decalcification before histological processing. Decalcification process is the removal of mineral from the bone tissue with the utilization of chemical agents in order to submit the tissue to histotechnical chelation in microtome.

The mineralization level of tissue bone directly influences decalcification protocols. It takes longer to decalcify bones with high mineral density. Time and the utilized chemical agent can damage the tissue either a lot or less, leading to quality loss in the cell analysis of different staining, immunohistochemistry, and/or molecular techniques.

In bone marrow biopsy, the fragment is delicate with trabeculae that are little thickened and in which the hematopoietic, adipose, and bone tissues are closely related and have different distribution patterns. In normal hematopoiesis or in disease setting, the bone marrow analysis depends on the evaluation of these components individually or together.

The biological nature of these elements is very different among themselves and, although in bone marrow biopsy the main aim is to analyze the hematopoietic tissue, its architectural relationship with the bone component takes an important place as well. Thus, the decalcification of this material is quite challenging since it is necessary to apply a technique that allows to cut bone trabeculae without influencing negatively the morphological, immunophenotypical, and molecular evaluations of the hematopoietic tissue.

There are different options available to perform this decalcification. The main examples are

- nitric oxide (NO_3_ 10%)
- formic acid 25% with sodium citrate
- hydrochloric acid (HCl 10%) associated with EDTA (ethylene diamine tetra acetic acid) and potassium sodium tartrate
- HCl 3.5%
- HCl 3.5% associated with EDTA and potassium sodium tartrate
- EDTA 10% in pH 8.0 and adjusted with sodium hydroxide

The targeted histological cut in the microtome is 3.0-millimicron thick. In general, the most successful decalcification protocols with the smallest cell damage for complementary exams are those that apply EDTA with hydrochloric acid.

Bone marrow aspirate clot (BMC) is an alternative to this methodology because it does not need decalcification. The clot allows the same kind of histological evaluation as bone marrow biopsy, except for the analysis of architectural relationship between parenchyma/bone. The hematopoietic tissue is enriched in the clot exam, allowing adequate cuts, utilization of several histochemical staining, and/or immunophenotypical evaluation and/or molecular analysis [5, 6]

When compared to BMB, BMC presents better antigenic expression in IHC techniques, mainly because it does not need decalcification procedure. Moreover, in situ hybridization techniques showed better results in BMC [5].

Although occasionally BMC isolated analysis is not enough to diagnose architectural alterations of bone marrow, its use results in complementary data to myelogram and BMB and may substitute the latter in cases of immediate unavailability of the exam, technical difficulty in accessing the bone, or regarding the patient's comfort if multiple evaluations are necessary for the follow-up of hematological diseases [3, 7]. Therefore, it is advantageous with histological characterization from a single anesthesia event and single bone marrow puncture.

Due to its complementarity with BMB, BMC increases sensitivity to diagnose hemopathies because it provides an additional site for anatomopathological analysis, which is especially useful for lymphoma staging [8]. Moreover, BMC allows histological evaluation and pathologist's opinion in clinical contexts in which BMB is not routinely done, such as in megaloblastic hemopathies and immune-mediated thrombocytopenic purpura [9]. Our service experience for more than 4 decades using BMC confirms the validity of this procedure for diagnosis, many times fortunate ones, which would be seen only in BMB (e.g., granulomas, lymphoid aggregates, necrosis focus, edema, and megakaryocyte clustering) [9].

Therefore, BMC represents a simpler technique without the need of additional analgesia but local anesthesia, in which a quick bone marrow aspirate offers the possibility of not only morphological evaluation but also anatomopathological analysis equivalent to BMB, speeding up the diagnosis and improving the patient's comfort.
